# The Algorithm of a Game-Based System in the Relation between an Operator and a Technical Object in Management of E-Commerce Logistics Processes with the Use of Machine Learning

**DOI:** 10.3390/s21155244

**Published:** 2021-08-03

**Authors:** Ryszard K. Miler, Andrzej Kuriata, Anna Brzozowska, Akram Akoel, Antonina Kalinichenko

**Affiliations:** 1 Faculty of Management and Finance, WSB University in Gdańsk, 80-266 Gdańsk, Poland; rmiler@wsb.gda.pl (R.K.M.); akuriata@wsb.gda.pl (A.K.); 2 Faculty of Management, Czestochowa University of Technology, 42-201 Częstochowa, Poland; annabrzozowskapcz@gmail.com; 3 The Briese Schiffahrts GmbH & Co. KG, Hafenstraße 12, 26789 Leer, Germany; akram.akoel@briese.de; 4 Institute of Environmental Engineering and Biotechnology, University of Opole, 45-040 Opole, Poland; 5 Department of Information System and Technology, Poltava State Agrarian Academy, 36003 Poltava, Ukraine

**Keywords:** e-commerce, machine learning algorithms, a game-based system, a logistics zero-sum game, Bayesian network

## Abstract

Machine learning (ML) is applied in various logistic processes utilizing innovative techniques (e.g., the use of drones for automated delivery in e-commerce). Early challenges showed the insufficient drones’ steering capacity and cognitive gap related to the lack of theoretical foundation for controlling algorithms. The aim of this paper is to present a game-based algorithm of controlling behaviours in the relation between an operator (OP) and a technical object (TO), based on the assumption that the game is logistics-oriented and the algorithm is to support ML applied in e-commerce optimization management. Algebraic methods, including matrices, Lagrange functions, systems of differential equations, and set-theoretic notation, have been used as the main tools. The outcome is a model of a game-based optimization process in a two-element logistics system and an algorithm applied to find optimal steering strategies. The algorithm has been initially verified with the use of simulation based on a Bayesian network (BN) and a structured set of possible strategies (OP/TO) calculated with the use of QGeNie Modeller, finally prepared for Python. It has been proved the algorithm at this stage has no deadlocks and unforeseen loops and is ready to be challenged with the original big set of learning data from a drone-operating company (as the next stage of the planned research).

## 1. Introduction: E-Commerce Development Ways and Challenges

The convenience, security and safety, and user experience of e-commerce have improved exponentially since its inception in the 1970s; however, its sense continues to change as new techniques, tools, and possibilities are gradually introduced. Unfortunately, this observation does not refer to the field of scientific e-commerce definitions and its range of understanding.

According to very first definitions from the beginning of modern electronic commerce by Roger Clarke, e-commerce is defined as the conduct of commerce in goods and services, with the assistance of telecommunication and telecommunication-based tools [1]. Jerry Allison also defines e-commerce as electronic contracting for the exchange of value through the use of information technology (IT), computing, and communication technology (ICT) [2]. According to Vladimir Zwass [3], e-commerce shares business information, maintaining business relationships and conducting business transactions by means of telecommunication networks. E-commerce has also been defined as doing business over the internet, selling goods and services that are delivered offline as well as products which can be “digitized” and delivered online, such as computer software. Anil Khurana defines e-commerce as using the power of computer, the internet, and shared software to send and receive product specifications and drawings; bids, purchase orders, and invoices; and any other type of data that needs to be communicated to customers, suppliers, employees, or the public. A similar but more developed definition is presented by Dan Cudjoe stating that e-commerce is “[…] business, technology, society, and skills of buying and selling of products and services with the aid of internet and computer or handheld and automated devices which involves the process of ordering products or services to the time of delivery to the consumer or customer” [4].

Unfortunately, there is a significant lack of definitions that reflect the newest “achievements” of e-commerce based on artificial intelligence (AI) implementation in the automation of transportation as a part of e-commerce supply/delivery processes [5]. This is relevant especially considering the hierarchical framework of e-commerce, consisting of three meta-levels: infrastructure, services, and products [6]. Additionally, several nodal problems have defined future developments in e-commerce, including integrating electronic platforms and electronic data interchange (EDI), new intermediation, and automation and structurally integrated autonomous transportation processes [7]. According to recent research findings, this is a way e-commerce can develop [8]. However, apart from a few attempts at the enlargement of e-commerce application to automated processes in warehousing [9] and management [10], there is a lack of developed definitions covering automated delivery processes (with use of AI/ML) as a vital and modern part of e-commerce.

Utilising this cognitive gap, the Authors make an attempt to prove that automation and autonomous transportation in e-commerce logistic processes have become practical activities and should be therefore treated as a part of contemporary e-commerce [11]. As a logic consequence, this type of e-commerce activity should be supported by the properly prepared ML algorithms with control applications (prepared in the field of applied theory) for better use of automated guided vehicles (AGV), including drones within the range of B2C e-commerce delivery processes [12].

The organization of this paper is based on the architecture of the presented research. The domain of the entire research is set on the modern e-commerce activities (depicted in the Introduction), and it assumes utilization of AI and ML in the field of e-commerce development and optimization (paragraph 2). The discussion (part 3) shows a cybernetic foundation of the selected elements of a game-based system (GS) and its usability in ML in the activities related to e-commerce automated delivery systems (drones). The solution is presented in paragraph 4. It involves application of algebraic methods viewed from the game-based perspective of e-commerce logistics processes (entities: OP/TO). The findings related to this part of the research are presented in paragraph 5 (Results): an original model of a game-based process as a logistics zero-sum game with an algorithm of choice optimization for e-commerce services has been developed. In order to verify the presented algorithm, a simulation based on a Bayesian Network has been introduced. A structured set of possible strategies (OP/TO) as a matrix (C) has been calculated and presented with the use of QGeNie Modeller, and finally the algorithm has been prepared with Python (only the initial dataset). The presented paper and this stage of research have been eventually concluded in paragraph 6. The research will be continued with the use of a big dataset (learning data) obtained from a drones-operating company (flights of unmanned aerial systems (UAS) beyond visual line of the operator’s sight (BVLOS) with full information about flight conditions e.g., weather, cargo, directions, battery status, geographical conditions, topography, etc.—the Authors’ note).

## 2. Artificial Intelligence and Machine Learning as Tools for E-Commerce Development

The past decade has been a time of unprecedented development of AI—including not only breakthrough research on ML algorithms [13] but also more common application of intelligent machines in various fields of human life [14]. Considering their widespread application in the pragmatics of economic processes, logistics processes that take place in e-commerce constitute an excellent testing field for the implementation of AI and ML solutions. ML(the term of machine learning (ML) was introduced in 1959 by Arthur Samuel who defined it as a field of study that gives computers the ability to learn without being explicitly programmed—the Authors’ note) is a branch of AI (the term was introduced by a computer scientist and a pioneer in the field of machine learning, Tom Mitchell—the Authors’ note) defined as “[…] developing and testing computer algorithms which allow computer software to improve through experience” [15]. ML is a method by which we expect to achieve AI. In practice, ML involves working with sets of data in order to find common and universal behaviour patterns, while deep learning (DL) [16] is based on multilayer perceptrons (MLPs) [17] understood as a mathematical function mapping some set of input values to output values [18].

According to Tom Mitchell [19], we can state that a machine learns how to perform a “T” task on the basis of experience “E” and a measure of performance quality “P” if the quality of performing the “T” task is improved with an increase in experience “E”, measured with “P”. Hence, the main aim of ML solutions is the practical application of algorithms to develop automatic systems that can improve themselves using experience (collected data) and, based on that, can acquire new knowledge.

ML can be classified by various criteria. Considering the type of examples and information provided [20], it is possible to distinguish supervised learning and unsupervised learning. Some representatives of the AI theoretical stream [21,22] indicate another method, namely, reinforcement learning.

In accordance with the assumed classification of ML methods, supervised learning can be observed when a set of data provided to a machine to learn also includes expected answers, whereas unsupervised learning takes place when there are no answers provided except for a set of data [23,24]. Reinforcement learning is observed when a system operates in a completely unknown environment. There are no specific input and output data. The only piece of information received by a learning machine is a reinforcement signal. The reinforcement signal can be either positive (reward) or negative (punishment)(an example can be a new game, the rules of which we do not know. After the game has been finished, we find out whether we have won or lost (reward or punishment). Hence, in the subsequent games, the level of the player’s advancement should be higher (as it results from the increase in experience)—the Authors’ note).

The algorithm of reinforcement learning can be generally defined as a repetitive (recursive) procedure of knowledge acquisition by the trial-and-error method. In applied terminology, in the control theory [25,26], it is possible to state that a controller (a regulator) interacts with a controlled object (environment or process), using three signals:

- The status of the system (x),
- Control (action–u),
- Reward–r (or control cost).

According to the assumed scheme of the procedures [27], at each stage of the algorithm, the controller observes the status of the object and then performs an action which takes the object to the next stage. At the same time, the controller receives a signal that evaluates (assesses) the performed action in the form of a reward. After the reward has been received, the controller takes the next step in the algorithm.

Understood in such a way, the model of procedures in reinforcement learning algorithms is applied in various systems, e.g., Game-based Systems (GS) (two-entity zero-sum games in the environment of the theory of logistics games), which can be utilized in e-commerce automated delivery systems [28].

## 3. Discussion: Selected Elements of a Game-Based System (GS) and Its Usability in ML in the Field Related to E-Commerce Automated Delivery Systems

Recently, the development of e-commerce has dramatically increased [29], especially considering the pandemic (covid-19) change in customer behaviours and a need for contactless logistic processes mainly in purchasing and delivery phases of such specific (adopted to the pandemic situation) chains of supply.

To achieve this goal, several innovative delivery techniques have been introduced and practically challenged. Apart from the wide use of AGV within logistic processes, there is an idea of automated drone delivery. Prime Air is a drone delivery service currently in development by Amazon. Operations were expected to begin in selected cities in late 2019; however, in December 2020 the service was still not available (Amazon received US Federal Aviation Administration approval to operate its fleet of Prime Air; however, it is not the only company seeking to expand commercial drone delivery; Alphabet-owned Wing and the US UPS have also been granted FAA approval, and overall status of the concept at the end of May 2021 is still experimental —the Authors’ note.)

This service is designed to use delivery drones to autonomously fly individual packages to customers (within e-commerce chain of supply) within 30 min from ordering. To be qualified for such a service, the order must be small and light enough (up to 5 pounds—2.25 kg) to fit the drone cargo box and delivery location must be within a 10-mile (16 km) radius of the participating Amazon order fulfilment centre. The biggest challenge is, however, to introduce an intelligent delivery drone control algorithm based on the game theory and use of AI within logistic e-commerce system.

Considering various approaches towards the definition of a system [30,31,32,33], it is possible to identify a category related to this term, which is referred to GS. Such systems, in which there are two entities participating in a game, can be theoretically and practically applied in the environment of logistics processes. The functioning of GS involves two types of information [34]:

- Indispensable initial information about the properties of the process (IIPP) (t∈θ), which is to form a set of parameters referring to the properties of the process implemented in (GS), which are necessary to identify a possible set of solutions and tasks of the convenience function (*fv*);
- Initial (a priori) information referring to the properties of the object (IIPO) (t∈θ), which will become a predefined set of the object parameters, information about limitation, coercion, and other factors indispensable to the implementation of the process in (GS).

Obtaining the IIPP and IIPO information also assumes the use of the Internet sources and big data [35]. It allows the object and the function of purpose to have an indispensable scope of information indicated. Hence, the information about the object includes:

- Information about the operator of the object
  - Information about external factors and interference affecting the object;
  - Information about the response of the object to signals.
- Information that defines the purpose includes
  - The criterion defining the quality of the (GS) operation,
  - The satisfying signal determining the purpose of operation;
- Working information WI (t∈θ), a set of information about the status of the process acquired.

An identifier of GS is using orders generated from the minimal piece of working information, according to the algorithm of playing the game (G). In accordance with the principles [36], the game consists of numerous subsequent stages, and it is reflected in a given game-based process. As a result, in GS orders are most often generated by one of the entities participating in the game. The finished game (G) is presented with the use of matrices [*M*]. A matrix is a function $f:\left\{1,2,\ldots ,\bar{m}\right\}\times [1,2,\ldots n\}\to R$ that is defined with the following equation [33]:

(1) $$f:\left(i,\;j\right)={\bar{m}}_{ij}$$

This function is explicitly defined by its values, which are presented as the following matrix [33]:

(2) $$\left[M\right]=\left[\begin{array}{c} {\bar{m}}_{11},\;\;{\bar{m}}_{1w},\;\cdots ,{\bar{\;m}}_{1n}\\ {\bar{m}}_{21},\;\;{\bar{m}}_{22},\;\cdots ,{\bar{\;m}}_{2n}\\ \cdots \;\;\;\;\;\;\;\;\;\;\;\;\;\;\;\;\;\;\;\;\;\;\;\;\;\;\;\;\;\;\;\;\\ \cdots \;\;\;\;\;\;\;\;\;\;\;\;\;\;\;\;\;\;\;\;\;\;\;\;\;\;\;\;\;\;\;\;\\ {\bar{m}}_{m1},\;\;{\bar{m}}_{m2},\;\cdots ,{\bar{\;m}}_{mn} \end{array}\right]$$

**Figure 1:**
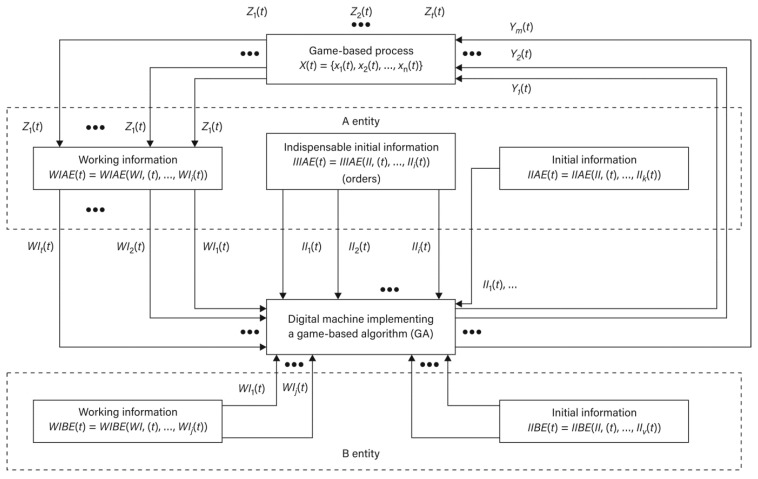
Information model of a game-based system (GS) [33].

The main principle of GS is selecting orders on the basis of a comparison performed in a set of solutions—possible choices at each stage of the process in the system. The criterion for comparing various possible solutions is the convenience function *fv*. This function is given during the development of the (GS), based on the analysis of the process. Raj, Biswas, and Srivastava [36] and Kordel and Kuriata [33] refer to the solutions that correspond to the extreme value *fv* as to quasi-optimal. The main link of a game-based system is a digital machine, which implements a game-based algorithm: it defines a set of possible choices and a quasi-optimal choice in that set. The game-based system (GS) and its essence are presented in Figure 1.

At the beginning of the process in a game-based system, some working information about the A entity is put into a digital machine implementing a game-based algorithm. Based on the working information and initial information (indispensable initial information), the digital machine defines a quasi-optimal choice and generates relevant orders. The orders affect the process implemented in (GS). The indispensable working information about action or about the status of the B entity at the initial moment (generating the first order) can be the complete IIPO (t∈θ).

The most important parameters of the dynamics of processes implemented in the (GS) can be expressed with the use of two basic characteristics: a time-varying value of the expected convenience function E(fv(t∈θ)) and the dispersion of the convenience function R(fv(t∈θ)) defined by the form of that function.

The sequence of the values of the expected convenience function E(fv(t∈θ)) at the particular stages of the operation is characterised by the main expected relation of the operation and its change in time. The discussed sequence is a discrete function of time that defines the expected value of the convenience function after the impact on the particular n-stages of the operation is finished. The process implemented in the (GS) is always a random process. The convenience function *fv* is also a discrete function of time. The second characteristic of the dynamics of the process implemented in the (GS) is a change in the dispersion of the function in time. The dispersion of the convenience function R(fv(t∈θ)) can be characterised by the entropy or the distribution of that function.

Based on the above-mentioned considerations and general statements [36,37], it is possible to state that the notion of a game-based system can be expressed with the use of its semantic model which is a set-theoretic model. Hence, a model of the (GS) notion can be the following system of notions [33]:

(3) $$GS=GS\{\{A(IIIAE(t),\;IIAE(t),\;WIAE(t);\;B(IIBE(t),\;WIBE(t);\;GA\{[M\},\;X(t)\}\;S_{A}\left(t\right);\;S_{B}\left(t\right);S_{A}^{*}\left(t\right);\;S_{A}^{*}\left(t\right);\;WW_{A}\left(t\right);\;WW_{A}\left(t\right);\;t\in \theta ;\;R\}\}$$

where *GA*—Is a game-based algorithm; *S_A_*(*t*)—is a set of possible strategies of the *A* entity in the (GS) at the particular moment (t∈θ); *S_B_*(*t*)—is a set of possible strategies of the *B* entity in the (GS) at the particular moment (t ∈ θ); $S_{A}^{*}\left(t\right)$—is a set of mixed strategies of the *A* entity in the (GS) at the particular moment (t ∈ θ); $S_{B}^{*}\left(t\right)$—is a set of mixed strategies of the *B* entity in the (GS) at the particular moment (t ∈ θ); $WW_{A}\left(t\right)$—is the level of the payoff obtained by the *A* entity if the *A* entity applies the $S_{A}^{i}\left(t\right)$ strategy and the *B* entity applies the $S_{B}^{j}\left(t\right)$ strategy in (GS); $WW_{B}\left(t\right)$—is the level of the payoff obtained by the *B* entity if the *B* entity applies the $S_{B}^{j}\left(t\right)$ strategy and the A entity applies the $S_{A}^{i}\left(t\right)$ strategy in the (GS); and *R*—is the set of relations observed among the elements in the (GS).

**Figure 2:**
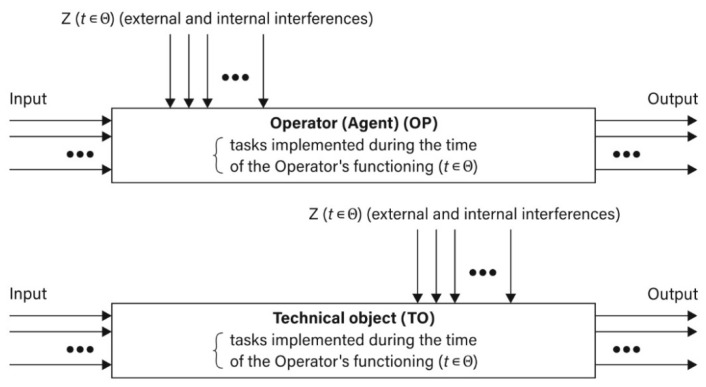
Information model (OP) and (TO) in the time (t∈Θ): adapted from [33].

Presented in the Equation (3), the structure of the (GS) may become a foundation for theoretical considerations including also logistics games. It may be also treated as a basis for decisions, which are comprised of a set of possible decisions and are made after the implementation of the process in a defined logistics system, for example, viewed from the perspectives of (OP) and (TO) in the particular time (t ∈Θ). The information model of such a relation is presented in Figure 2.

Provided above, the presentation of the (GS) relation for (OP) and (TO) in the time makes it (t∈Θ) possible to present the processes that can be observed there in more detail, through the algebraisation [38] of a game-based system in the relation between an operator and a technical object with the use of matrices, Lagrangian functions, systems of differential equations, and set-theoretic notations, with the assumption that the considerations refer to the theory of logistics games.

## 4. Solution: Application of Algebraic Methods Viewed from the Game-Based Perspective of E-Commerce Logistics Processes (Entities: An Operator (OP)—A Technical Object (TO) e.g., a Drone and Its Operator)

**Figure 3:**
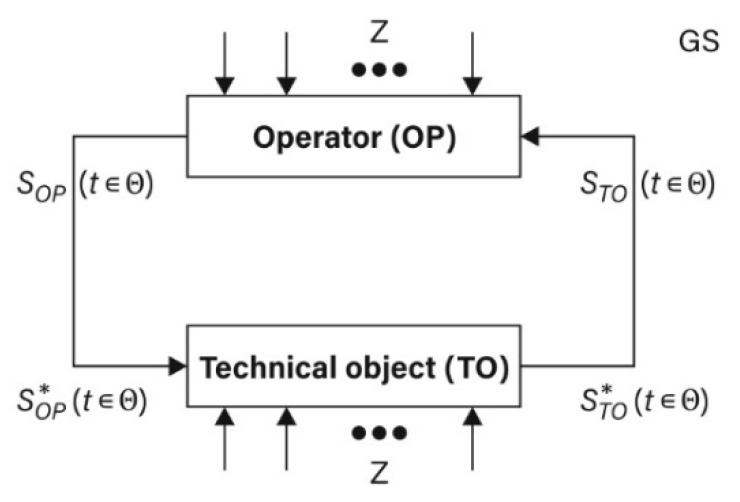
Process-based approach toward the (GS) for (OP) and (TO) during the time for a (t∈Θ) logistics game (LG), adapted from [33].

A problem to be considered is a description of the course of a process under the conditions of uncertain information between two logistics-oriented entities: an operator (an agent) (OP) and a technical object (TO), which makes a drone and its operator a very suitable example. The course of the process must include potential sets of strategies for both entities (S_OP_, S_TO_) and the level of interferences (Z) defined under the IPSA (t∈Θ). A process-based approach toward the discussed system for the reality of a logistics game (LG) is presented in Figure 3.

Hence, it is possible to assume that the LG is a sum of elements, which match the following equation [33]:

(4) $$LG=LG\{\left(OP\right),\left(TO\right);\;\left(G\right);S_{OP}\left(t\right);\;S_{TO}\left(t\right);S_{TO}^{*}\left(t\right);\;S_{TO}^{*}\left(t\right);\;WW_{OP}\left(t\right);\;WW_{TO}\left(t\right);\;t\in \theta ;\;R$$

A finished game (G) is given with the use of the matrices:

(5) $$G\left(i,j\right)\in {\left\{1,2,\;\ldots ,\;n\right\}}^{2}\to c_{ij}\in C$$

where $\left(i,\;j\right)=\bar{1,\;4\;}\;,\;$ because it is assumed that the sets of possible strategies of (TO) and of (OP) have four elements each, hence: 
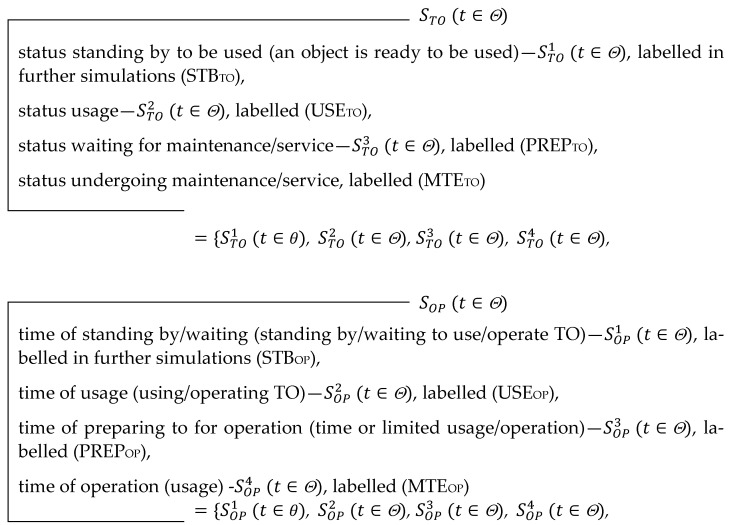


Considering a statement referring to the game theory [39], it is assumed that each two-entity zero-sum game has at least one solution that defines the value of the game and the entities’ optimal strategies if:

- α=ν=β—It is a closed game with optimal pure strategies for the (OP) and (TO) sub-systems in the (GS);
- α≤ν≤β—It is an open game and sets of mixed strategies for the (OP) and (TO) sub-systems in the (GS).

The sets of mixed strategies for the (OP) and (TO) sub-systems can be presented as follows [33]:

(6) $$S_{OP}^{*}\;\left(t\right)=\left(\begin{array}{c} S_{OP}^{1}\;\left(t\right),S_{OP}^{2}\;\left(t\right),\;S_{OP}^{3}\;\left(t\right),\;S_{OP}^{4}\;\left(t\right)\;\\ p_{OP}^{1}\;\left(t\right),p_{OP}^{2}\;\left(t\right),\;p_{OP}^{3}\;\left(t\right),\;p_{OP}^{4}\;\left(t\right) \end{array}\right)\;\;S_{TO}^{*}\;\left(t\right)=\left(\begin{array}{c} S_{TO}^{1}\;\left(t\right),S_{TO}^{2}\;\left(t\right),\;S_{TO}^{3}\;\left(t\right),\;S_{TO}^{4}\;\left(t\right)\;\\ q_{TO}^{1}\;\left(t\right),q_{TO}^{2}\;\left(t\right),\;q_{TO}^{3}\;\left(t\right),\;q_{TO}^{4}\;\left(t\right) \end{array}\right)\;$$

where: $p_{OP}^{1}\;\left(t\right),p_{OP}^{2}\;\left(t\right),\;p_{OP}^{3}\;\left(t\right),\;p_{OP}^{4}\;\left(t\right)$—is the frequency of selection from the set of possible strategies for the (OP) sub-system in the (GS) during the time period t∈θ, $q_{TO}^{1}\;\left(t\right),q_{TO}^{2}\;\left(t\right),\;q_{TO}^{3}\;\left(t\right),\;q_{TO}^{4}\;\left(t\right)$—is the frequency of selection from the set of possible strategies for the (TO) sub-system in the (GS) during the time period t∈θ, and:

(7) $$p_{TO}^{1}\;\left(t\right),p_{TO}^{2}\;\left(t\right),\;p_{TO}^{3}\;\left(t\right),\;p_{TO}^{4}\;\left(t\right)=1$$

(8) $$q_{TO}^{1}\;\left(t\right),q_{TO}^{2}\;\left(t\right),\;q_{TO}^{3}\;\left(t\right),\;q_{TO}^{4}\;\left(t\right)=1$$

and, as a consequence [33]:

$\overset{4}{\underset{i=1}{\sum }}c_{i1}p_{OP}^{i}$—Is the expected value of the gain of the (OP) sub-system in the (GS) if the (TO) sub-system in the (GS) selects the first strategy $S_{OP}^{1}\;\left(t\right)$ in the time period t∈θ,
$\overset{4}{\underset{i=1}{\sum }}c_{i2}p_{OP}^{i}$—Is the expected value of the gain of the (OP) sub-system in the (GS) if the (TO) sub-system in the (GS) selects the second strategy $S_{OP}^{2}\;\left(t\right)$ in the time period t∈θ,
$\overset{4}{\underset{i=1}{\sum }}c_{i3}p_{OP}^{i}$—Is the expected value of the gain of the (OP) sub-system in the (GS) if the (TO) sub-system in the (GS) selects the third strategy $S_{OP}^{3}\;\left(t\right)$ during the time period t∈θ,
$\overset{4}{\underset{i=1}{\sum }}c_{i4}p_{OP}^{i}$—Is the expected value of the gain of the (OP) sub-system in the (GS) if the (TO) sub-system in the (GS) selects the fourth strategy $S_{OP}^{4}\;\left(t\right)$ in the time period t∈θ. 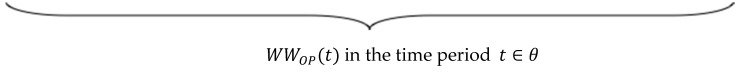 and:
$\overset{4}{\underset{j=1}{\sum }}c_{j1}q_{TO}^{i}$—Is the expected value of the loss of the (OP) sub-system in the (GS) if the (TO) sub-system in the (GS) selects the first strategy $S_{TO}^{1}\;\left(t\right)$ in the time period t∈θ;
$\overset{4}{\underset{j=1}{\sum }}c_{2j}q_{TO}^{i}$—Is the expected value of the loss of the (OP) sub-system in the (GS) if the (TO) sub-system in the (GS) selects the second strategy $S_{TO}^{2}\;\left(t\right)$ in the time period t∈θ;
$\overset{4}{\underset{j=1}{\sum }}c_{3j}q_{TO}^{i}$—Is the expected value of the loss of the (OP) sub-system in the (GS) if the (TO) sub-system in the (GS) selects the third strategy $S_{TO}^{3}\;\left(t\right)$ in the time period t∈θ;
$\overset{4}{\underset{j=1}{\sum }}c_{4j}q_{TO}^{i}$—Is the expected value of the loss of the (OP) sub-system in the (GS) if the (TO) sub-system in the (GS) selects the fourth strategy $S_{TO}^{4}\;\left(t\right)$ in the time period t∈θ.

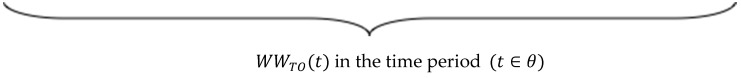


The expected value of the gain of the (OP) sub-system in the (GS) in the time period t∈θ is defined as:

(9) $$\left(\overset{4}{\underset{i=1}{\sum }}c_{i1}p_{OP}^{i}\right)\;q_{TO}^{1}\left(t\right)+\left(\overset{4}{\underset{i=1}{\sum }}c_{i2}p_{OP}^{i}\right)\;q_{TO}^{2}\left(t\right)+\left(\overset{4}{\underset{i=1}{\sum }}c_{i3}p_{OP}^{i}\right)\;q_{TO}^{3}\left(t\right)+\left(\overset{4}{\underset{i=1}{\sum }}c_{i4}p_{OP}^{i}\right)\;q_{TO}^{4}\left(t\right)$$

The expected value of the loss of the (OP) sub-system in the (GS) in the time period t∈θ is defined as:

(10) $$\left(\overset{4}{\underset{j=1}{\sum }}c_{ij}q_{TO}^{1}\right)p_{OP}^{i}\left(t\right)+\left(\overset{4}{\underset{j=1}{\sum }}c_{j2}q_{TO}^{i}\right)p_{OP}^{2}\left(t\right)+\left(\overset{4}{\underset{j=1}{\sum }}c_{3j}q_{TO}^{i}\right)p_{OP}^{3}\left(t\right)+\left(\overset{4}{\underset{j=1}{\sum }}c_{i4}q_{TO}^{j}\right)p_{OP}^{4}\left(t\right)$$

In order to define the value of the game (v), to identify the optimal strategy from a set of possible strategies of the (OP) sub-system in the (GS) and the (TO) sub-system in the (GS) and to define the level of the payoff, it is necessary to [33]

- Identify the values $p_{TO}^{1},p_{TO}^{2},\;p_{TO}^{3},\;p_{TO}^{4}$ and the values $q_{TO}^{1},q_{TO}^{2},\;q_{TO}^{3},\;q_{TO}^{4}$, for which the value of the expected gain of the (OP) sub-system in the (GS) is maximal with the use of the Lagrangian function [40]:
  (11) $$L\left(p_{OP}^{1},p_{OP}^{2},\;p_{OP}^{3},\;p_{OP}^{4},q_{TO}^{1},q_{TO}^{2},\;q_{TO}^{3},\;q_{TO}^{4};\lambda_{1},\lambda_{2},\right)=\left(\overset{4}{\underset{i=1}{\sum }}c_{i1}p_{OP}^{i}\right)q_{TO}^{1}+\left(\overset{4}{\underset{i=1}{\sum }}c_{i2}p_{OP}^{i}\right)q_{TO}^{2}+\;\left(\overset{4}{\underset{i=1}{\sum }}c_{i3}p_{OP}^{i}\right)q_{TO}^{3}+\left(\overset{4}{\underset{i=1}{\sum }}c_{i4}p_{OP}^{i}\right)q_{TO}^{4}+\lambda_{1}\left(p_{OP}^{1},p_{OP}^{2},\;p_{OP}^{3},\;p_{OP}^{4}-1\right)+\lambda_{2}\left(q_{TO}^{1},q_{TO}^{2},\;q_{TO}^{3},\;q_{TO}^{4}-1\right)$$
  where $\lambda_{1},\;\lambda_{2}$ are the Lagrangian multipliers, and
- Equate the partial derivatives to zero, obtaining the following [33]:
  (12) $$\{\begin{array}{c} \frac{\partial L}{\partial p_{OP}^{1}}=0\\ ..........\\ ..........\\ \frac{\partial L}{\partial p_{OP}^{4}}=0 \end{array}\;\;\;\{\begin{array}{c} \frac{\partial L}{\partial q_{TO}^{1}}=0\\ ..........\\ ..........\\ \frac{\partial L}{\partial q_{TO}^{4}}=0 \end{array}\;\;\{\begin{array}{c} \frac{\partial L}{\partial \lambda_{1}}=0\\ ..........\\ ..........\\ \frac{\partial L}{\partial \lambda_{2}}=0 \end{array}$$
- Calculate the values $q_{TO}^{1},q_{TO}^{2},\;q_{TO}^{3},\;q_{TO}^{4}$ and $p_{TO}^{1},p_{TO}^{2},\;p_{TO}^{3},\;p_{TO}^{4},$ for which the expected value of the loss of the (TO) sub-system in the (GS) reaches its minimum with the use of the Lagrangian function described with the Equation (11) and so:
  (13) $$L\left(q_{TO}^{1},q_{TO}^{2},\;q_{TO}^{3},\;q_{TO}^{4};p_{OP}^{1},p_{OP}^{2},\;p_{OP}^{3},\;p_{OP}^{4},\lambda_{1},\lambda_{2},\right)=\left(\overset{4}{\underset{j=1}{\sum }}c_{1j}q_{TO}^{1}\right)p_{OP}^{1}+\;(\overset{4}{\underset{j=1}{\sum }}c_{2j}q_{TO}^{1})p_{OP}^{2}+\left(\overset{4}{\underset{j=1}{\sum }}c_{3j}q_{TO}^{1}\right)p_{OP}^{3}+\left(\overset{4}{\underset{j=1}{\sum }}c_{4j}q_{TO}^{1}\right)p_{OP}^{4}+\lambda_{1}\left(q_{TO}^{1}+q_{TO}^{2}+q_{TO}^{3},+q_{TO}^{4}-1\right)+\lambda_{2}\left(p_{OP}^{1},p_{OP}^{2},\;p_{OP}^{3},\;p_{OP}^{4}-1\right)$$
- To equate the partial derivatives to zero (in a way analogical to the system of Equation (12)).

## 5. Results: An Original Model of a Game-Based Process as a Logistics Zero-Sum Game with an Algorithm of Choice Optimisation for E-Commerce Services

**Figure 4:**
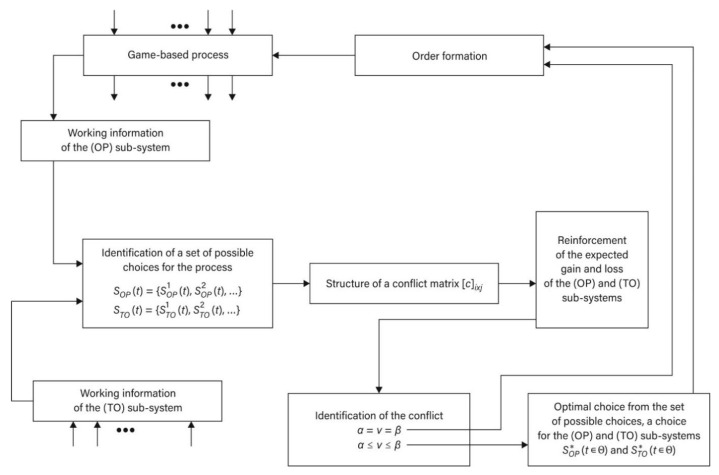
Model of a game-based process as a logistics game (LG) in the game-based system (GS) for the relation of the (OP) and (TO) during the time period (t∈Θ): adapted from [33].

**Figure 5:**
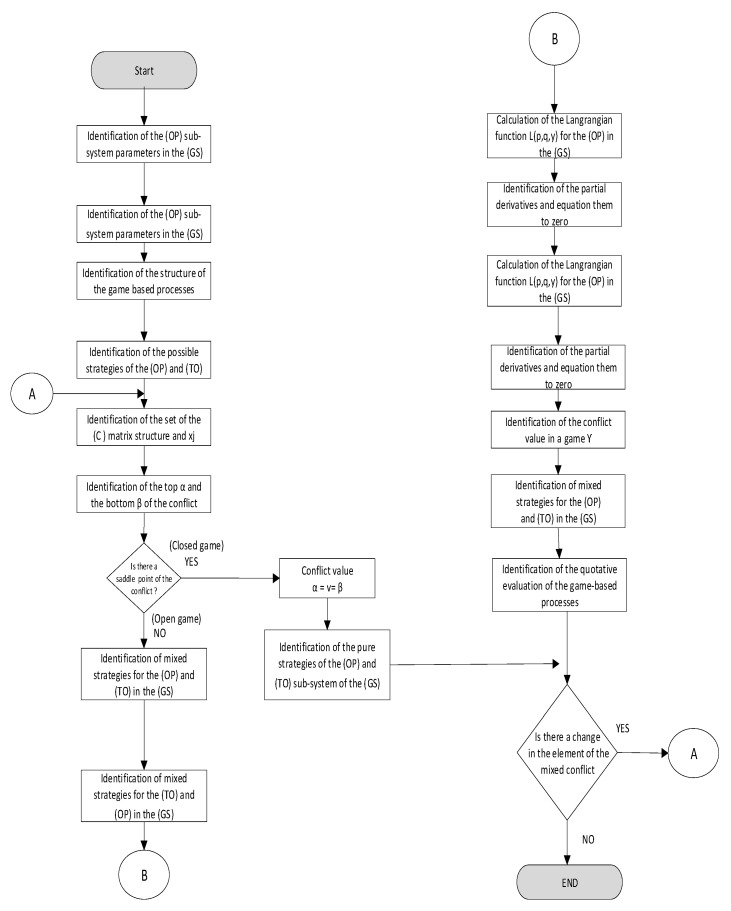
Algorithm applied to search for optimal strategies in the set of possible strategies of the (OP) and (TO) sub-systems in the game-based system (GS), adapted from [33].

The above-mentioned considerations allow the Authors to present:

- A model of a game-based process as an approach toward the theory of logistics games (LG) in the game-based system (GS), as presented in Figure 4;
- An algorithm applied to search for optimal strategies in a set of possible strategies in the (OP) and (TO) sub-systems in the game-based system (GS), as presented in Figure 5.

In order to verify the presented algorithm, a simulation based on a Bayesian network has been developed. Considering data availability, only an experimental (reduced) dataset has been used; however, all the variables have been discretized and selected for the Bayesian network structure learning and parameter learning, according to the BN rules. The structured set of possible strategies (OP/TO) as a matrix (Cxj) has been calculated and presented in Table 1.

**Figure 6:**
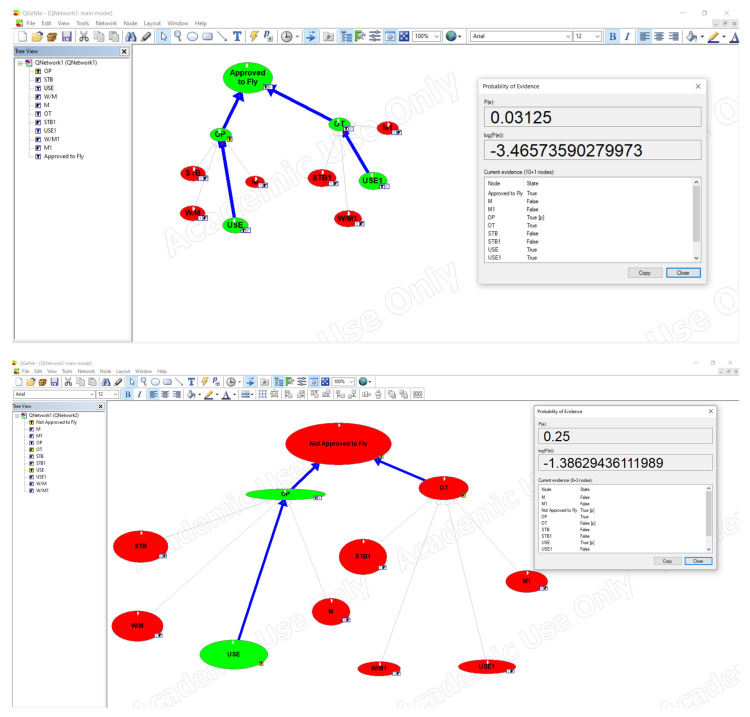
Selected results (optimization of possible strategies (OP) and (TO) from the matrix (Cxj)) with use of QGeNie Modeller.

Aiming to achieve the maximum level of simplicity at this stage, all the preferences excluding diagonal of the matrix have been calculated as zero. All the attributes are discrete, and there is no missing value. A 10-fold cross-validation method has been employed in this procedure, and selected results (optimization of possible strategies) are depicted with use of QGeNie Modeller in Figure 6. 

**Figure 7:**
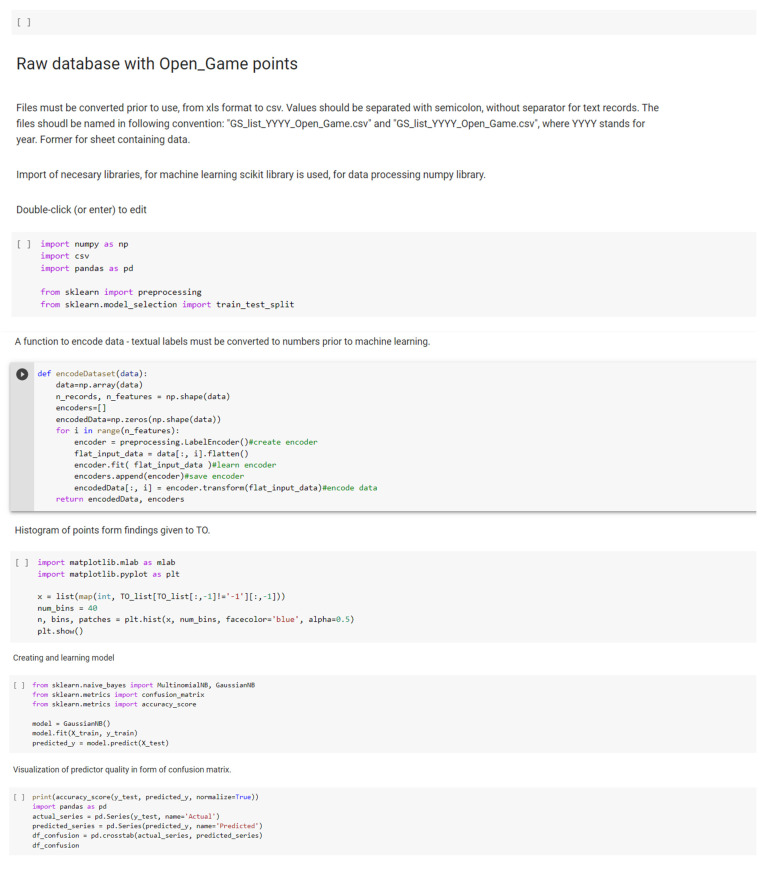
Proposed algorithm (selected elements) prepared for Python (without real data).

Finally, the algorithm has been prepared for Python (only the initial dataset) as illustrated in Figure 7. 

The entire research has been planned in two stages: the first one, as concluded in this article, has been based on theoretical foundation for developing an optimizing algorithm, and it has been proved that the algorithm at this stage has no deadlocks and unforeseen loops and is ready to be challenged with the original learning data from drones-operating company (as the second stage of the research).

Considering the pragmatics of the entity operating drones, based on the matrix of the strategy C x, j, in the first stage of the research an answer is provided to the following question: which drone of the whole fleet can be approved to fly, and which drone is not approved to fly because of its technical condition, remaining minimal battery range, and some maintenance procedures it has to undergo? The second stage of the research will provide answers to more complicated questions related to optimisation because in the fleet of drones having operational capabilities, each drone has different parameters related to its operation time (remaining battery capacity). The optimisation task at this stage is to assign a drone with a task (a delivery flight) in which the existing capabilities can be used in the most efficient way. Each flight covers a different distance, flight conditions can change (temperature, wind direction, and altitude), cargo weight may vary (maximum 2.5 kg), or other parameters that determine the consumption of energy from the battery during the performance of the task may vary (so, only one drone can be the most efficient one to perform a particular task; the system should handle such choices for at least 100 drones simultaneously). The Authors hope that the suggested algorithm will allow the interested parties to considerably increase the optimisation level in decisions they have to make and will also allow entities that use drones to deliver goods within the automated e-commerce supply chain to improve their operational and economic efficiency.

The development of the algorithm presented above has been critical, considering the purpose function of the scientific proof, and it has also allowed the Authors to draw general conclusions.

## 6. Conclusions: The Value of the Discussed Model for the Algorithmisation of E-Commerce Logistics Processes

The use of opportunities related to ML in logistics processes taking place in e-commerce supply chains can, first of all, provide effectiveness, efficiency, reliability, and reduction of costs generated by processes and their control (management) systems.

Therefore, there has been a continuous search for solutions that can not only collect all information coming from a logistics process but also provide its detailed analysis in real time. Algorithmisation of processes can be applied to achieve that aim. It is dedicated to their further application in control (management) systems that use ML elements. Specific to e-commerce logistics processes (B2C automated delivery), the way in which the OP-TO relation functions in the GS (as one of the fundamental models of logistics-oriented behaviour and processes which may refer both to control and management aspects in supply chains) has been analysed and mathematically proved. Algorithmisation of GS processes has allowed the Authors to develop a model of a game-based process in a GS, with consideration of the theory of LG. It has also been possible for the Authors to develop an algorithm applied to search for optimal strategies in an identified set of possible solutions in the OP and TO sub-systems for GS (based on possible automated drone delivery). 

**Figure 8:**
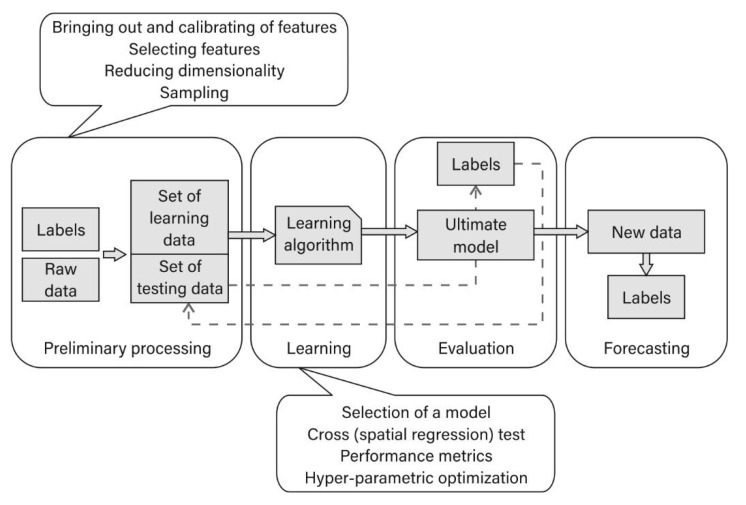
Predictive modelling, adapted from [22].

Considering the fact that there are various types of algorithms that describe logistics processes and that can be applied in ML (classification algorithms, regression algorithms, and grouping algorithms), it should be indicated that the results of the research and the scientific proof presented in this paper may be used in further work on development of an applicative ML regression algorithm for the GS OP-TO. In practice, it may transpire that it is necessary to provide further predictive modelling based on a sequence of actions presented in Figure 8. This should accelerate the introduction of pragmatic processes involved in automated drone delivery in e-commerce chains of supply.

The results of the research, the review of scientific literature [41,42,43,44,45,46,47], and business studies [48] allow the Authors to draw more general conclusions on advantages related to the use of ML algorithms in pragmatics of control and management of logistics processes, which are summarized below. When considered from a synthetic perspective, the following statements can be assumed to be true:

- ML algorithms are able to establish priorities and to automate the process of making managerial decisions (also in the context of control) in complex and simple logistics systems (e.g.,: OP-TO);
  ML algorithms are able to establish priorities and to automate the process of making managerial decisions (also in the context of control) in complex and simple logistics systems (e.g.,: OP-TO);
- ML uses historical and real-time generated data for learning; this fact defines flexibility of management systems that apply ML;
- Algorithm-based business uses advanced ML algorithms to achieve a high level of automation—transition to this type of activity makes way for new innovative business models;
- ML provides the possibility to analyse large resources of complex data and streaming data and to draw conclusions—also from predictive analysis—that can be unavailable to the human mind;
- Intelligent, ML-supported business processes can considerably increase efficiency of logistics-oriented systems; they make it possible to develop precise plans and forecasts, to automate tasks, to reduce costs, and to eliminate most human errors;
- As there is an increasing interest in the development of ML systems to include a function for explainability, it may be further developed in cognitive computing systems.

To sum up, ML based on GS algorithms can help to achieve better business results in the field of logistics, starting with initiating proper action, based on new chances and risk factors and culminating in the ability to make precise forecasts of results before costly and consequential e-commerce supply-chain decisions are made. Focused on more pragmatic processes associated with automated and autonomous transportation as a part of e-commerce chains of supply/delivery, ML can significantly improve the learning curve and radically accelerate real and wide implementation of such a solution into the market. As has been already mentioned, the continuation of the stage 2 and future research is needed to develop and test further predictive modelling to optimize e-commerce supply-chain management. The early adopters of ML for supply-chain logistics will be able to exponentially grow their efficiency and productivity. Hence, their superior supply (and production) will quickly outperform other outdated operations and will shut down the more inefficient and costlier outdated competitors.

## Figures and Tables

**Table 1:** Matrix (Cxj) set of possible strategies (OP) and (TO).

		(OP)			
		STB	USE	PREP	MTE
(TO)	STB	1	0	0	0
	USE	0	1	0	0
	PREP	0	0	1	0
	MTE	0	0	0	1

## Data Availability

Not applicable.

## Acronym

Sets and Graphs, Indexing, linear Algebra Operations, Probability and Game Theory, Functions, Datasets and Distribution:

*A*	A set
*R*	The set of real numbers
{0, …, *n*}	The set containing all integers between 0 and *n*
[*a*, *b*]	The real interval including *a* and *b*
*a_i_*	Element *i* of *a*
*M_ij_*	Element *i*, *j* of the M matrix
*det(A)*	Determinant of *A*
*P(a)*	A probability distribution over a discrete variable
*p(a)*	A probability distribution over a continuous variable
*M^T^*	Transpose of the M matrix
(t∈θ)	Time calculated in given timeframe
(t∈Θ)	The particular time
*λ*_1_, *λ*_2_	The Lagrangian multipliers
*f*(*x*; *θ*)	A function *x* parametrized by *θ*; often *f(x)* argument *θ* is omitted to lighten notation
*f*: *A*→*B*	The function f with domain *A* and range *B*
*a*⊥*b*	The random variables *a* and *b* are independent
*fv*	The convenience function
*X*	A set of training examples
*x^(i)^*	The *i*th example (input) from a dataset
*y^(i)^*	The target associated with *x^(i)^* for supervised learning
*S*	Set of strategies
